# The Persistence of Slowed Time Experience During the COVID-19 Pandemic: Two Longitudinal Studies in France

**DOI:** 10.3389/fpsyg.2021.721716

**Published:** 2021-09-01

**Authors:** Sylvie Droit-Volet, Natalia Martinelli, Johann Chevalère, Clément Belletier, Guillaume Dezecache, Sandrine Gil, Pascal Huguet

**Affiliations:** ^1^Université Clermont Auvergne, CNRS, LAPSCO, Clermont-Ferrand, France; ^2^Université de Poitiers et CNRS, CeRCA, Poitiers, France

**Keywords:** COVID-19, lockdown, time, longitudinal studies, boredom, emotion, rhythm of life, sleep

## Abstract

The home confinement imposed on people to fight the COVID-19 pandemic interrupted the flow of time by disrupting daily life, making them feel that time was passing slowly. The aim of this longitudinal study was to evaluate the evolution over time of this subjective experience of time and its significant predictors (boredom, decreased happiness, life rhythm, and sleep quality). Twso samples of French participants were followed up: the first for several weeks during the first lockdown (April 2020) and then 1year later (April 2021; Study 1), and the second during the first lockdown (April 2020) and then 6months (November 2020) and 1year later (April 2021; Study 2). Our study shows that the French participants have the feeling that time has passed slowly since the beginning of the first lockdown and that it has not resumed its normal course. This is explained by a persistent feeling of boredom characteristic of a depressive state that has taken hold in the population. The findings therefore suggest that the repeated contexts of confinement did not contribute to re-establishing a normal perception of time, to which a subjective acceleration of time would have testified.

## Introduction

In 2020, the worldwide coronavirus pandemic (COVID-19) led the governments of many countries, including France, to adopt severe measures requiring the home confinement of the population. In France, an 8-week total lockdown was imposed from March 17 to May 10. However, the epidemic has not been stopped. Further waves have emerged, with a high rate of hospital admissions and many people requiring artificial respiration. To cope with the pressure on the healthcare system, the French government decided to re-confine the population during each wave while waiting for a significant proportion of the population to be vaccinated: during the second wave, from October 30 to December 15, 2020 (for 6 1/2weeks; T1), and the third wave, from April 3 to May 2, 2021 (for 4weeks; T2), i.e., just 1year after the first lockdown. However, the measures became decreasingly severe from one lockdown to the next, with 1h of the authorized absence from home (shopping for basic necessities, physical activity, and dog’s needs) per day at a distance of 1km from home for the first lockdown, 3h at 20km for the second, and all day long until 9p.m. at 10km for the third. Universities remained closed, but schools were open during the second and third lockdowns. In addition, the third lockdown included 15days of school vacation (10–24 April) with no distance restriction. Many French newspapers even questioned the notion of confinement for the third lockdown: “It’s not really a confinement” (e.g., Berrod, 2021).

The first home confinement interrupted the flow of time for individuals by disrupting their daily life. This disruption of time flow produced significant time distortions. During the first lockdown, people did indeed report a feeling that the speed of time passage had slowed down compared to before, regardless of their age and sex. This slowing down was clearly observed in France (Droit-Volet et al., 2020; Martinelli et al., 2021a; Study 1 and Study 2), Italy (Cellini et al., 2020), and the United Kingdom (Ogden, 2020).

Subsequently, the authors of these studies tried to identify the factors related to this change in subjective experience of time passage during the lockdown by asking their participants numerous questions about their living conditions (e.g., celibacy and living space), activities (physical activity and activity load), feelings (e.g., fear, anger, sadness, boredom, and happiness), or their life quality (e.g., sleep quality, life rhythm, and life satisfaction). They also assessed their mental health using validated scales to estimate their levels of depression, anxiety, stress, impulsivity, and alexithymia. The results revealed that living conditions, stress, anxiety, and fear – including the fear of being infected and sick – had little or no effect on changes in time experience (Droit-Volet et al., 2020; Ogden, 2020; Martinelli et al., 2021a). Instead, it was the emotions linked to the difficulty of life during confinement and the disruption of the life rhythm due to the cessation of normal social life that had an influence. Indeed, the feeling of a slowing down of time was found to be significantly associated with a decrease in life satisfaction due to the lack of social interaction (Ogden, 2020) and a reduced level of happiness (Droit-Volet et al., 2020; Martinelli et al., 2021a). A powerful feeling of boredom was also found to be associated with the increase in the feeling that time was passing slowly. The boredom is defined by Eastwood et al. (2012) as a negative mood associated with a lack a satisfying activity when individuals are unable to successfully attract attention with internal (thoughts) or external (environmental stimuli) information. Cellini et al. (2020) and Martinelli et al. (2021a) also observed the important role of the disturbance of the regularity of the life rhythm associated with the impairment of sleep quality. Indeed, people reported that they found it more difficult to sleep due to their new rhythm of life, and the worse the quality of sleep was judged to be, the more slowly time seemed to pass. Of course, the feelings of happiness and boredom, sleep quality, and life rhythm were significantly related. The more difficult people found it to sleep, the less energy they reported they had, and the more bored and the less happy they were. However, the statistical analyses revealed that each of these four factors (boredom, happiness, sleep quality, and life rhythm) accounted for a part of the variance in the individual time judgments, although the self-reported level of boredom explained the greatest part (Droit-Volet et al., 2020; Martinelli et al., 2021a). In particular, boredom and sadness are feelings typical of depressed people, and these COVID-19 studies also found greater time distortions in the most depressed individuals, although the correlation between time and depression scores remained low.

According to the theory of the passage of time, the subjective experience of time mirrors conscious processes related to the introspective analysis of our internal state in a specific context, i.e., changes in self-duration related to context (Droit-Volet, 2018; Droit-Volet and Dambrun, 2019; Droit-Volet and Martinelli, in press; Martinelli and Droit-Volet, 2021). To reuse the metaphor proposed by Holman and Grisham (2020, p. 63), “time is the window through which we see our lives unfold.” And when life is not going well, time stretches. However, people theoretically have the capacity to overcome this adversity and rebound (Yip et al., 2021). The COVID-19 pandemic and the lockdown measures were described as a storm: a violent and brutal one. But after the storm, people may have developed strategies to cope with the new situation. We can assume that the reactions to new life circumstances disrupted the flow of time and its perception, but that the latter improved over time because people adapted to it. It is therefore assumed that there was an improvement in time judgment (acceleration of the passage of time) and the underlying factors (boredom, happiness, sleep, and life rhythm) over the course of the first lockdown and the following months. However, to clarify whether this improvement (if it indeed exists) is not simply due to individuals’ non-compliance with the public lockdown measures, participants’ degree of compliance with these measures was also assessed. Consequently, we followed up the participants for several weeks during the first lockdown and also 6 and 12months later. There were subsequent periods of confinement, but with less restrictive measures, as described above.

An alternative explanation is that despite the months that have passed and made it possible to recover from a stressful episode and despite the relaxation of the constraints of home confinement, restrictive directives are nevertheless still present (i.e., home confinement, quarantine, and restricted social interaction). It is therefore possible that, in the French population, the slowing down of the passage of time has remained the same, bringing with it the same negative emotions (boredom and low level of happiness). This is the conclusion of the recent and only longitudinal study, which was conducted by Ogden (2021) in the United Kingdom over a period of 8months. However, the rhythm of life and the quality of sleep should necessarily improve to some extent due to individuals’ adaptation to the new living conditions and the less restrictive lockdown measures, even if depressed people often present sleep disorders (for a review, Fang et al., 2019). A dissociation between the emotional state and the real conditions of life could indicate the long-term establishment of a negative mood, involving a chronic and growing depression, that would result in a still disturbed perception of time. In this case, the feelings observed in the COVID-19 studies during the first lockdown would not represent an episodic state in reaction to a new situation but rather a true trauma. According to recent studies, the COVID-19 pandemic and the home confinement constitute a real traumatic experience leading to post-traumatic stress (e.g., Bonsaksen et al., 2020; Cooke et al., 2020). Traumatic situations can indeed create the sense that time has stopped or slowed down (Holman et al., 2016). Therefore, in our study, we included a post-traumatic scale. Furthermore, as reported by Lewin (1942), traumatic situations prevent people from having goals, from projecting themselves into the future. Traumatic events lead people to remain focused either on the past trauma or on the present trauma (current pain and management of current constraints) without daring to consider the future. In all cases, there is a loss of sense of the future, thus increasing the risk of depression (Abramson et al., 1989; Beck and Weishaar, 1989). During the COVID-19 pandemic, the studies using the time perspective scale indicated that people were less future-oriented (Sobol et al., 2020; Virna and Brahina, 2020; Wu et al., 2021), and the less future-oriented individuals tended to be more depressed (Wu et al., 2021). The depressed individuals also tended to be less compliant with public health measures (Sobol et al., 2020; Martinelli et al., 2021b). However, women appeared to be more focused on the future than men during the COVID-19 pandemic (Sobol et al., 2020; Virna and Brahina, 2020). Consequently, we also measured our participants’ degree of focus on the past, future, and present and its relationship with the judgment of time.

We conducted two longitudinal studies with two different samples of French participants. The first sample was followed up several times during the first lockdown (April 2020) and 1year after (April 2021; Study 1). The second was surveyed three times: (1) during the first lockdown (April 2020), (2) 6months after (November 2020), and (3) 1year after (April 2021; Study 2). Although they were questioned during a lockdown period each time, the measures successively became less restrictive from one lockdown to the next. The survey concerned the passage of time and the extent to which it was determined by various previously identified factors: Emotions, quality of sleep and regularity of the life rhythm, and psychopathological measures (depression, anxiety, and social isolation; Martinelli et al., 2021a). In addition, three new measures were included in order to reflect the longitudinal aims of the investigation: temporal focus, trauma scale, and the participants’ compliance with the lockdown measures.

## Study 1

### Method

#### Participants

The Research Ethics Committee of the University Clermont Auvergne approved this study (IRB00011540-2020-31), which was conducted in accordance with the principles of the Declaration of Helsinki. The participants were given a form explaining that they could stop the current survey at any time and decide to not complete the following surveys. Their anonymity was guaranteed and their data hosted on the university’s local server. The initial sample was composed of 1,330 French adults who completed the survey once (1,009 women and 321 men; *M*_age_=40.98, *SD*_age_=16.38, *M*_Education year_=14.83, and *SD*_Education year_=2.99). The same participants were surveyed 1year later, with a loss of 65%, i.e., 469 participants (358 women and 111 men; *M*_age_=44.65, *SD*_age_=16.49, *M*_Education year_=15.851, and *SD*_Education year_=2.91). The participants were also followed up five times during the first lockdown after the first survey with a loss of participants at each survey going from 73 to 86%: *N*_T1_=356, *N*_T2_=495, *N*_T3_=430, *N*_T4_=364, and *N*_T5_=186. Due to the loss of participants, we have reported in Table 1 the results of the first survey (T0) for the initial sample (T0_Initial-sample_ *N*=1,330) and those for the participants who responded at least twice to the survey during the first lockdown (T0_2-completed-surveys_ *N*=681). Some of the data of the initial sample at T0 were published in Martinelli et al. (2021a). The original data therefore concerned the results obtained from T1 to T5 and T1year (April 2021).

**Table 1:** Mean (standard deviation) of age and scores for the different survey times in Study 1.

	T0_Initial-sample_		T0^b^		T1		T2		T3		T4		T5		T1year	
	April 2020		Lockdown periods (April–May 2020)												April 2021	
	*M*	*SD*	*M*	*SD*	*M*	*SD*	*M*	*SD*	*M*	*SD*	*M*	*SD*	*M*	*SD*	*M*	*SD*
Age^a^	40.98	16.38	40.40	16.45	39.26	16.06	40.12	16.54	40.66	16.71	40.80	16.58	41.47	17.78	44.65	16.50
Time before lock	5.52	1.30													5.13	1.37
Time during lock	4.60	1.57	4.49	1.44	4.60	1.46	4.63	1.48	4.88	1.43	4.85	1.40	4.83	1.39	4.44	1.62
Boredom	2.93	1.90	2.45	1.73	2.48	1.71	2.74	1.78	2.42	1.64	2.42	1.68	2.47	1.70	3.11	2.03
Sleep	4.47	1.83	4.36	1.82	4.54	1.88	4.65	1.82	4.76	1.80	4.79	1.78	4.63	1.91	4.43	1.79
Life rhythm	4.66	1.91	4.75	1.83	5.11	1.79	5.07	1.76	5.25	1.63	5.24	1.68	5.24	1.61	5.24	1.66
Happiness	4.48	1.46	4.59	1.49	4.73	1.33	4.60	1.26	4.67	1.28	4.71	1.29	4.78	1.19	4.21	1.52
Anger	3.05	1.84	2.12	1.54	2.18	1.49	2.24	1.49	2.27	1.54	2.13	1.51	2.14	1.47	3.11	1.80
Fear	3.21	1.74	2.47	1.58	2.31	1.46	2.46	1.46	2.32	1.42	2.41	1.44	2.48	1.46	2.89	1.54
Anxiety	3.54	1.85	2.88	1.75	2.79	1.61	2.92	1.58	2.84	1.60	2.84	1.55	2.86	1.61	3.72	1.76
High arousal	3.41	1.68	3.18	1.65	3.51	1.55	3.57	1.55	3.81	1.55	3.84	1.56	3.85	1.54	3.10	1.54
Low arousal	3.96	1.63	4.24	1.63	4.23	1.49	4.25	1.41	4.09	1.50	4.24	1.49	4.24	1.48	3.54	1.48
Compliance	5.65	0.73	5.46	0.85	5.29	1.00	5.34	0.95	5.27	0.95	5.20	1.01	5.23	0.95		
Isolation	19.78	5.11	19.37	5.37	25.76	6.70	24.26	9.01	23.23	9.92	22.92	10.09	24.91	7.65	20.57	5.42
Depression	4.92	4.98													5.85	5.39
Anxiety	12.95	4.33													12.93	4.54
PT stress															14.28	13.78
Past focus															14.46	5.10
Future focus															15.19	5.10
Present focus															18.06	4.78

a *N*_T0Initial-sample_=1,330 (1,009 women and 321 men); lockdown periods: *N*_T0_=681, *N*_T1_=356, *N*_T2_=495, *N*_T3_=430, *N*_T4_=364, and *N*_T5_=186; and *N*_T−1year_=469 (358 women and 111 men).

b Results of participants with at least two completed surveys during the first lockdown periods.

### Procedure

The participants completed the online LimeSurvey-based questionnaire seven times, i.e., six times during the total lockdown of the French population from March 17 to May 10, 2020 (T0, T1, T2, T3, T4, and T5), at a rate of one survey per week, and once 1year later in April 2021 during the third lockdown (T1year). As explained above, the government measures were severe for the first lockdown (teleworking, 1h of authorized absence from home per day within 1km from home, and closure of universities and schools) and very mild in the third lockdown (absence authorized all day until 9p.m. within 10km radius of home, schools open).

The questions were similar for the five “follow-up” surveys sent during the lockdown period. They took between 5 and 10min to complete. Among the questions, there was one on the subjective experience of the passage of time and a question for each factor previously identified as a predictor of experienced time (for more details on the questionnaire, see Martinelli et al., 2021a). These factors were emotions of boredom and happiness, quality of sleep, and regularity of the life rhythm. The participants’ responses for the other emotion questions (anger, fear, anxiety, high arousal, and low arousal) were also asked but only for additional information (see Table 1 for data). We also added a question about the participants’ compliance with the lockdown measures: “since the confinement was imposed, I have respected it and have gone out only rarely.” For all these questions, a 7-point Likert scale going from one to seven was used, i.e., from *very slow* to *very fast* (time), from not *at all* to *a lot/completely* (emotion, sleep, and life rhythm) and from *strongly disagree* to *strongly agree* (lockdown compliance). In addition, at each survey, we assessed the participants’ social isolation score by using the short version of the UCLA loneliness scale (S-UCLA; Russell, 1996). The participants’ depression and anxiety scores were also assessed at T0 and T1year using the Beck Depression Inventory (BDI; Beck et al., 1961; *α*_T0_=0.83, *α*_T−1year_=0.86), and the short form of the State-Trait Anxiety Inventory (S-STAI; Marteau and Bekker, 1992; *α*_T0_=0.87, *α*_T−1year_=0.88). For the last survey (T1year), we added a 20-item Post-traumatic Stress Disorder Checklist Scale for DSM-5 (PCL-5; Weathers et al., 2013; Ashbaugh et al., 2016; *α*=0.95) and the 12-item Temporal Focus Scale (TFS; Shipp et al., 2009; *α*=0.74), which includes four items per each loading factor of the scale: past, current, and future focus.

### Results and Discussion

#### Changes in Time Judgment and Its Predictors 1Year After the First Lockdown

**Figure 1:**
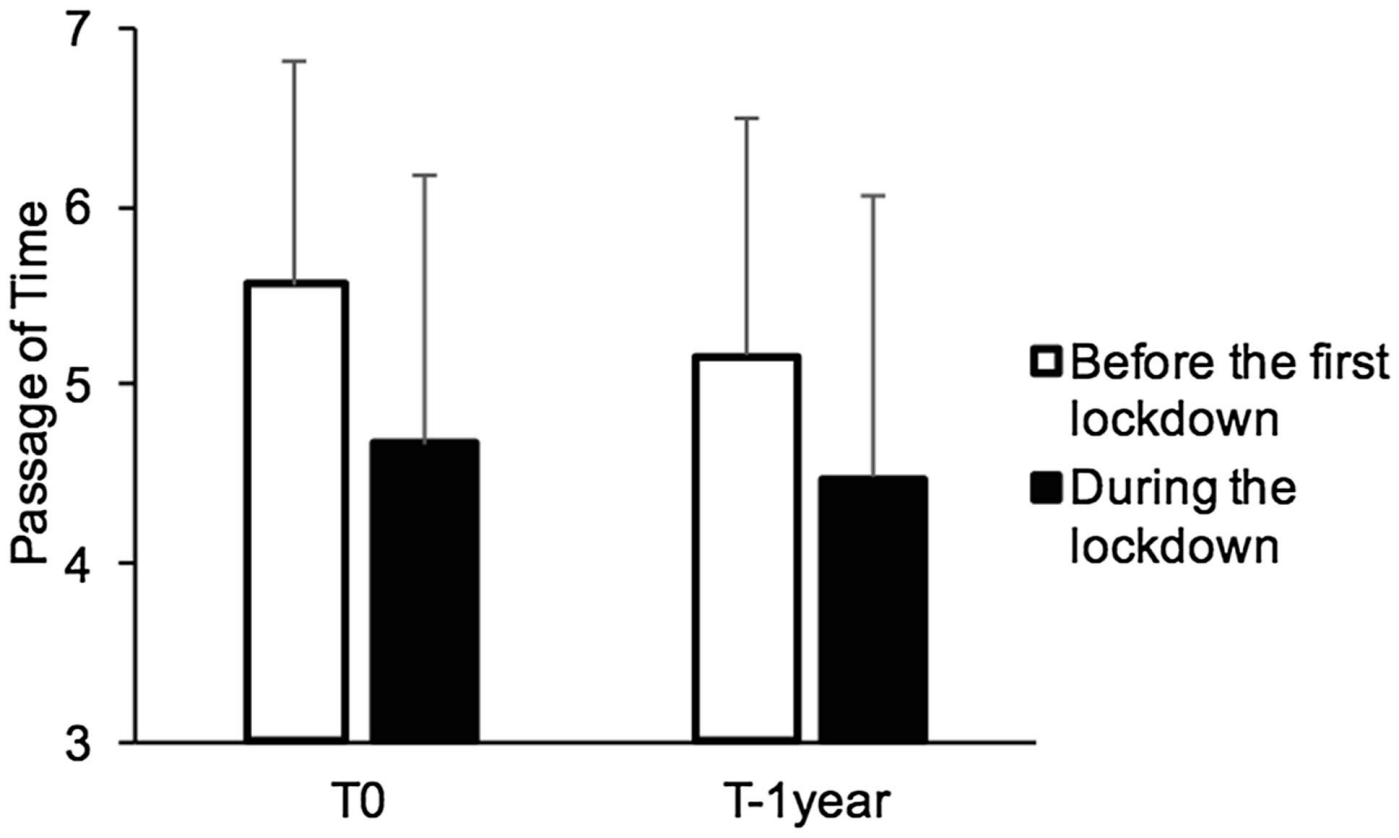
Mean ratings of passage of time for before and during the first lockdown (T0) and for one year after (T-1 year).

Figure 1 shows the judgment of experienced time during two periods, i.e., during the first lockdown (T0) and 1year later (T1year), and that of time as remembered from before the first lockdown. An ANOVA with two within-subjects factors (T0/T1year, before/during) was performed on time judgments. The ANOVA showed a significant main before/during effect, *F* (1, 395)=134.57, *p*<0.001, $\eta_{p}^{2}$=0.254, indicating a strong feeling that time slowed down during the lockdown (*M*_during_=4.575, *SD*=1.56) compared to the flow of time reported for life before the lockdown (*M*_before_=5.36, *SD*=1.30). There was also a significant main effect of the period, *F* (1, 395)=18.90, *p*<0.001, $\eta_{p}^{2}$=0.05, suggesting the establishment of a long-term sensation of a slowing down of time regardless of the changing lockdown conditions (*M*_T0_=5.12, *SD*=1,39; *M*_T–1year_=4.82, *SD*=1.46). The interaction between these two factors just reached significance, *F* (1, 395)=3.75, *p*=0.05, $\eta_{p}^{2}$=0.009. Indeed, there was little change in experienced time between T0 and T1year, suggesting that there was no recovery over time [*M*_T0_=4.67, *SD*=1.52; *M*_T1year_=4.48, *SD*=1.59, *t* (395)=1.93, *p*=0.055, Cohen’s *d*=0.102]. In addition, between T0 and T1year, there was a distortion in the remembered passage of time before the first lockdown, with this being rated as slower 1year afterwards [*M*_T0_=5.56, *SD*=1.26; *M*_T1year_=5.16, *SD*=1.34, *t* (395)=4.97, *p*<0.001, *d*=0.853], as if the participants’ focus on their current difficulties affected their judgment of remembered time. Indeed, the scores on the temporal perspective scale indicated that the participants were more focused on the present (*M*=18.06, *SD*=4.78) than on the past (*M*=14.46, *SD*=5.10) or the future [*M*=15.19, *SD*=5.10; *t* (404)=9.38, *p*<0.001, *d*=0.47; *t* (404)=8.42, *p*<0.001, *d*=0.42, respectively], but more on the future than the past, *t* (404)=8.42, *p*<0.001, *d*=0.11.

Along with a continued disrupted sense of time, the participants reported an increase in negative affects 1year after the first lockdown [boredom, *M*_T0_=2.8, *SD*=1.84, *M*_T1year_=3.07, *SD*=2.003, *t* (399)=−2.40; *p*=0.017, *d*=0.12; happiness, *M*_T0_=4.59, *SD*=1.43, *M*_T1year_=4.22, *SD*=1.49, *t* (399)=4.67; *p*<0.001, *d*=0.24; depression score, *M*_T0_=4.63, *SD*=4.45, *M*_T1year_=5.67; *SD*=5.23, *t* (357)=−4.09; *p*<0.001, *d*=0.22], with an increased feeling of social isolation [*M*_T0_=19.77, *SD*=4.97, *M*_T1year_=20.63, *SD*=5.45, *t* (349)=−3.24, *p*=0.001, *d*=0.18]. Only the level of anxiety did not change [*M*_T0_=12.77, *SD*=4.26, *M*_T1year_=12.95, *SD*=4.58, *t* (349)=−0.80, *p*<0.001, *d=* 0.04]. On the other hand, the rhythm of life had improved by being more regular [*M*_T0_=4.81, *SD*=1.87, *M*_T–1year_=5.26; *SD*=1.66, *t* (399)=−4.44, *d*=0.22] but without significant self-reported changes in sleep quality [*M*_T0_=4.47, *SD*=1.84, *M*_T–1year_=4.41; *SD*=1.78, *t* (399)=0.56, *d*=0.03].

To further examine the relationships between the individual changes over time in judgments of experienced time and changes in the other factors, we calculated difference scores between T1year and T0 for each variable and analyzed their correlations. As shown in Table 2, the growth of the feeling of a slowing down of time over a period of 1year was significantly associated with an increase in the levels of boredom (*R*=−0.32, *p*=0.001), sadness (decreased happiness; *R*=0.28, *p*<0.001), depression (*R*=−0.22, *p*=0.001), and social isolation (*R*=−0.16, *p*=0.003). In contrast, the time flow appeared to accelerate again with the return of a regular life rhythm (*R*=0.14, *p*=0.005) and improved sleep (*R*=0.13, *p*=0.008).

**Table 2:** Correlation matrix in Study 1.

S. No.		1	2	3	4	5	6	7	8	9	10	11	12
1.	Time^a^	1	−0.32^**^	0.13^**^	0.14^**^	0.28^**^	−0.16^**^	−0.22^**^	-,150^**^	−0.15^**^	−0.11^*^	0.07	0.15^**^
2.	Boredom	−0.32^**^	1	−0.12^*^	−0.17^**^	−0.41^**^	0.27^**^	0.39^**^	0.17^**^	0.12^*^	0.11^*^	−0.03	−0.13^*^
3.	Sleep	0.13^**^	−0.12^*^	1	0.22^**^	0.22^**^	−0.18^**^	−0.22^**^	−0.25^**^	−0.12^*^	-,106^*^	−0,02	0,04
4.	Life rhythm	0.14^**^	−0.17^**^	0.22^**^	1	0.19^**^	−0.12^*^	−0.19^**^	−0.09	−0.04	0.04	−0.01	0.07
5.	Happiness	0.28^**^	−0.41^**^	0.22^**^	0.19^**^	1	−0.40^**^	−0.56^**^	−0.31^**^	−0.21^**^	−0.21^**^	0.05	0.26^**^
6.	Isolation	−0.16^**^	0.27^**^	−0.18^**^	−0.12^*^	−0.39^**^	1	0.42^**^	0.34^**^	0.25^**^	0.26^**^	−0.07	−0.15^**^
7.	Depression	−0.22^**^	0.39^**^	−0.22^**^	−0.19^**^	−0.56^**^	0.42^**^	1	0.42^**^	0.35^**^	0.20^**^	0.01	−0.24^**^
8.	Anxiety	−0.15^**^	0.17^**^	−0.25^**^	−0.09	−0.31^**^	0.34^**^	0.42^**^	1	0.26^**^	0.15^**^	−0.01	−0.201^**^
9.	PT stress	−0.15^**^	0.12^*^	−0.12^*^	−0.04	−0.21^**^	0.25^**^	0.35^**^	0.26^**^	1	0.43^**^	−0.02	−0.30^**^
10.	Past focus	−0.11^*^	0.11^*^	−0.11^*^	0.04	−0.21^**^	0.26^**^	0.20^**^	0.15^**^	0.43^**^	1	0.21^**^	−0.22^**^
11.	Future focus	0.07	−0.03	−0.02	−0.01	0.05	−0.07	0.01	−0.01	−0.02	0.21^**^	1	0.04
12.	Present focus	0.15^**^	−0.13^*^	0.04	0.07	0.26^**^	−0.15^**^	−0.24^**^	−0.20^**^	−0.30^**^	−0.22^**^	0.04	1

a T−1year score−T0 score for all variables except for PT stress, past, future, and present focus.

* *p*<0.05;

** *p*<0.01

However, as in Martinelli et al. (2021a), the strongest correlation continued to be that between experienced time and the feeling of boredom. We thus conducted a linear hierarchical regression to identify the relevant predictors of changes in the time judgment between T1year and T0. In this regression analyses, the difference indexes (T1year score−T0 score) were used for all the considered factors. The results presented in Table 3 clearly show that boredom was the only reliable predictor of changes in experienced time even when the other factors (life rhythm, sleep, happiness, depression, and isolation) were entered into the equation. The order of factors in the models did not change the results. In summary, rather than an improvement, there was a maintenance of or slight increase in the distortion of experienced time which was explained by an increase in boredom, a negative affect, over a period of 1year.

**Table 3:** Hierarchical regression analysis on the changes (T_−_1year score−T0 score) in the judgment of the passage of time in Study 1.

S. No.		*B*	*SE*	*β*	*R* ^2^
**Model 1^a^**					
1.	Boredom	−0.262	0.042	−0.32	0.11^***^
Model 2					
1.	Boredom	−0.25	0.042	−0.31^***^	
2.	Life rhythm	0.083	0.047	0.09	
*Overall significance*			0.11^***^		
**Model 3**					
1.	Boredom	−0.244	0.042	−0.30^***^	
2.	Life rhythm	0.068	0.048	0.07	
3.	Sleep	0.082	0.053	0.08	
*Overall significance*			0.12^***^		
**Model 4**					
1.	Boredom	−0.208	0.046	−0.26^***^	
2.	Life rhythm	0.054	0.049	0.06	
3.	Sleep	0.066	0.053	0.07	
4.	Happiness	0.133	0.069	0.11	
*Overall significance*			0.13^***^		
**Model 5**					
1.	Boredom	−0.203	0.047	−0.25^***^	
2.	Life rhythm	0.053	0.049	0.06	
3.	Sleep	0.063	0.053	0.06	
4.	Happiness	0.114	0.077	0.10	
5.	Depression	−0.013	0.024	−0.04	
*Overall significance*			0.13^***^		
**Model 6**					
1.	Boredom	−0.203	0.047	−0.25^***^	
2.	Life rhythm	0.053	0.049	0.06	
3.	Sleep	0.063	0.054	0.06	
4.	Happiness	0.114	0.078	0.10	
5.	Depression	−0.013	0.025	−0.04	
6.	Isolation	1.92E-05	0.022	0.01	
*Overall significance*			0.13^***^		

a Difference index used for all the variables (T_−_1year score−T0 score).

*** *p*<0.001

**Figure 2:**
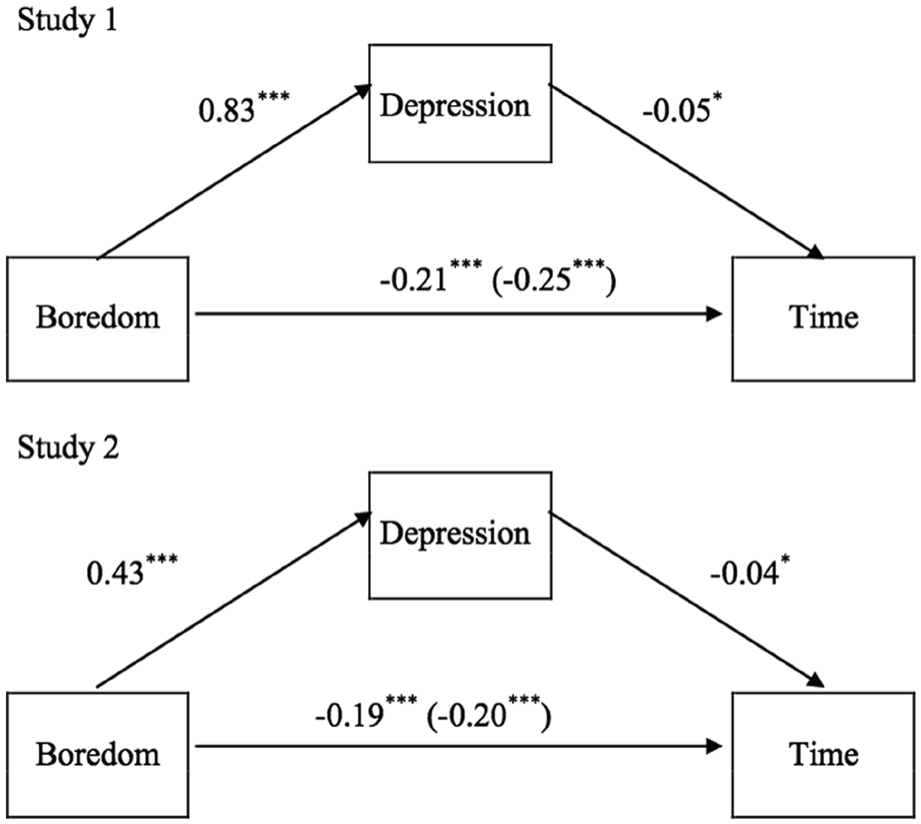
Mediation models for study 1 and study 2, showing the effect of boredom on time judgment with depression as a mediator.

Obviously, the increase in the level of boredom between T0 and T1year was highly correlated with the increase in the depression scores (*R*=0.39, *p*<0.001). Moreover, the increase in the depression scores between T0 and T1year was higher in individuals with higher post-traumatic stress scores (*R*=0.35, *p*=0.001), and the more individuals suffered from post-traumatic stress, the more they were focused on the past (*R*=0.43, *p*=0.001). The mediation analysis (Figure 2) revealed that the increase in the depression scores was indeed a significant mediator of the link between the changes in boredom and those in time perception (*E*=−0.0377, *SE*=0.018, 95%CI [−0.07, −0.003], *Z*=−2.10, *p*=0.036). However, the indirect effect of depression scores on time judgments remained small (15.1%) compared to the direct effect of boredom (84.9%, *E*=−0.21, *SE*=0.045, 95%CI [−0.30, −0.12], *Z*=−4.71, *p*<0.001). The scores on the post-traumatic scale or the temporal focus scale did not significantly mediate the effect of boredom on time judgment (all *p*s>0.05).

#### Changes in Time Judgment and Its Predictors Over the Duration of the First Lockdown

**Figure 3:**
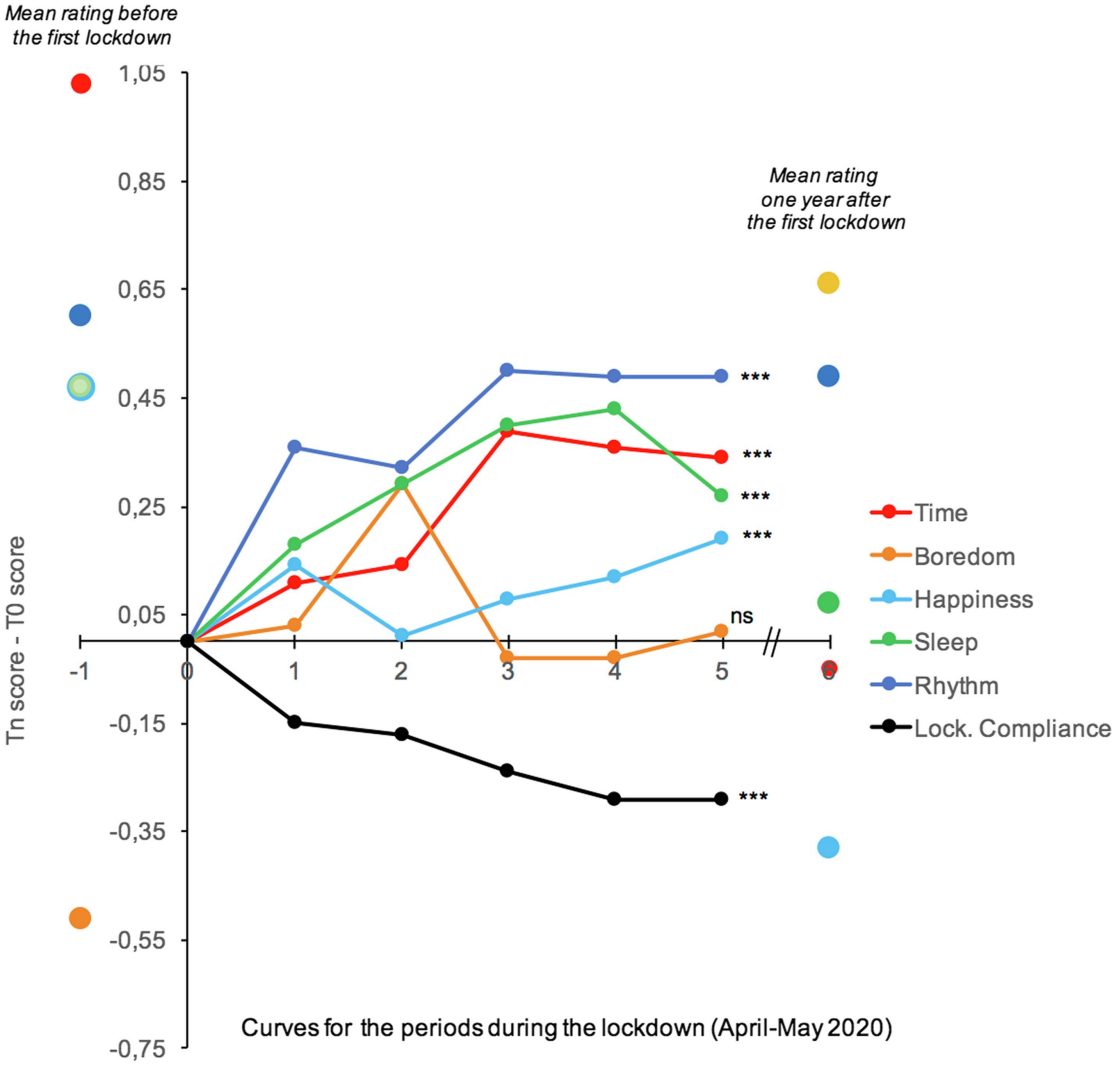
Evolution of difference index (Tn score - T0 score) for our variables of interest during the first lockdown (weekly measured). The score before the first lockdown and one year after is also given as a benchmark only.

Figure 3 shows the changes in time judgment and the other tested variables throughout the first lockdown, i.e., from T0 to T5. The mean scores before the first lockdown and 1year after is given in Figure 3 as a benchmark only. As suggested by Figure 3, although the rating score remained low and never reached the initial pre-lockdown level, there was a slight but significant improvement in the rating of time (time passed faster), as well as that of the other variables, except for boredom, which remained at a low floor level. When we performed linear regressions on each variable using the lockdown periods from T0 to T5 as factor and the participants as random effect, we did indeed find a significant lockdown period effect on time judgment (*E*=0.094, *SE*=0.0127, 95%CI [0.069, 0.119], *t*=7.30, *p*<0.001). As confinement continued, there was therefore a slight (*Δ*<1 on a 7-point scale) but significant improvement in time judgment, with time being judged to pass faster as the period of lockdown lengthened. In parallel, the participants tended to feel happier as lockdown progressed (*E*=0.0289, *SE*=0.01197, 95%CI [0.0054, 0.0524], *t*=2.42, *p*<0.001), but their level of boredom always remained low (*E*=−0.0013, *SE*=0.0161, 95%CI [−0.0303, 0.0329], *t*=0.08, *p*=0.93). More significantly, the participants reported that they slept better at the end of lockdown (*E*=0.0779, *SE*=0.0176, 95%CI [0.0043, 0.1124], *t*=4.42, *p*<0.001) and that their life rhythm was more regular (*E*=0.09034, *SE*=0.014, 95%CI [0.0625, 0.1182], *t*=6.37, *p*<0.001). However, the participants also reported that they were less compliant with the lockdown rules as the confinement at home lengthened (*E*=−0.0734, *SE*=0.007, 95%CI [−0.087, −0.0594], *t*=−10.30, *p*<0.001) and that they suffered more and more from isolation, as indicated by their scores on the social isolation scale (*E*=0.078, *SE*=0.096, 95%CI [0.592, 0.971], *t*=8.10, *p*<0.001).

To identify the best predictors of changes in time judgment over the entire lockdown period, we therefore performed a series of regressions on the time judgment with two factors: the lockdown period and each of the variables taken separately (lockdown compliance, boredom, happiness, life rhythm, and sleep). The participants were always used as random effect. The lockdown compliance effect and the lockdown compliance×lockdown period interaction were no longer significant (*p*>0.05), while the other factors remained significant (all *ps*<0.05). Therefore, the reduced compliance with the confinement measures did not explain the changes in time judgments over the lockdown period. Consequently, we included the various significant factors (boredom, happiness, life rhythm, and sleep) in the same regression model. The statistical model only revealed a boredom x lockdown period interaction (*E*=−0.2889, *SE*=0.119, 95%CI [−0.522, −0.0554], *t*=−2.42, *p*=0.015), indicating that the improvement in experienced time (increased speed of time) during the confinement period was related to a decrease in the level of boredom as the lockdown progressed.

## Study 2

### Method

#### Participants

A new sample of participants were followed up three times for a full year. The initial sample was composed of 1,082 people (3% of the participants did not respond to our questions of interest; 550 women and 532 men, *M*_age_=46.75, *SD*_age_=15.02, *M*_Education year_=12.88, and *SD*_Education year_=3.15). Out of these participants, 72.64% (*N*=786; 392 women and 394 men, *M*_age_=48.41, *SD*_age_=14.61, *M*_Education year_=12.87, and *SD*_Education year_=3.14), and 58.4% (*N*=632; 324 women and 308 men, *M*_age_=49.22, *SD*_age_=14.31, *M*_Education year_=12.76, and *SD*_Education year_=3.19) completed the second and the third surveys, respectively. Our surveys were always conducted in accordance with the principles of the Declaration of Helsinki and were approved by the Research Ethics Committee of the University Clermont Auvergne (MRA-19/20-18,372). Participants were recruited by a survey company (Easy panel) in return for a gift voucher. Their anonymity was guaranteed and their data hosted on the university’s local server.

### Procedure

The questionnaire was similar to that used in Study 1. However, in Study 2, the participants completed the questionnaire three times, once during each of the three lockdown periods imposed on the French population: at T0 from April 24 to 28, 2020, at T1 from November 12 to 18, 2020, and at T2 from April 6 to 12, 2021. As previously explained, the lockdown measures were severe, moderate, and mild at T0, T1, and T2, respectively. Some of the data at T0 have already been published (Martinelli et al., 2021a). The original data are therefore those for T1 and T2 and were compared to T0. As in Study 1, the questions of interest were those on the subjective experience of the passage of time and those related to its significant predictors (boredom, happiness, quality of sleep, and regularity of the life rhythm; Martinelli et al., 2021a), with an additional question on compliance with the lockdown measures. The participants’ responses for the other tested emotions (sadness, anger, fear, anxiety, high arousal, and low arousal) were reported for additional information only (Table 4). At each survey, we also assessed the individuals’ scores on depression (BDI), anxiety (S-STAI), and social isolation (S-UCLA). The reliability of these scales was satisfactory (BDI: *α*_T0_=0.89, *α*_T1_=0.90, *α*_T2_=0.90; S-STAI: *α*_T0_=0.90, *α*_T1_=0.89 *α*_T2_=0.90; and S-UCLA: *α*_T0_=0.80, *α*_T1_=0.80, *α*_T2_=0.79). Post-traumatic stress (PCL-5) and temporal focus were also assessed at the third time (*α*_T2_=0.97, *α*_T2_=0.87, respectively).

**Table 4:** Mean (standard deviation) of age and scores for the different survey time in Study 2.

	T0		T1		T2		Linear *F*-value	*Value of p*
	April 2020		November 2020		April 2021			
	*M*	*SD*	*M*	*SD*	*M*	*SD*		
Age^a^	46.75	15.02	48.41	14.61	49.22	14.31		
Time before lock	5.2	1.33	4.66	1.43	4.73	1.39	55.69	< 0.001
Time during lock	4.21	1.56	4.30	1.45	4.31	1.45	1.06	= 0.31
Boredom	3.46	1.88	3.32	1.80	3.34	1.80	0.98	= 0.32
Sleep	4.21	1.76	4.47	1.68	4.44	1.65	12.20	< 0.001
Life rhythm	4.53	1.76	5.08	1.62	4.99	1.53	27.60	< 0.001
Happiness	4.10	1.48	4.23	1.43	4.23	1.41	9.06	= 0.003
Anger	3.54	1.83	3.47	1.78	3.46	1.77	0.86	= 0.36
Fear	3.90	1.77	3.56	1.71	3.42	1.67	68.96	< 0.001
Anxiety	3.91	1.75	3.82	1.71	3.69	1.70	18.16	< 0.001
High arousal	3.26	1.58	3.17	1.46	3.30	1.49	0.58	= 0.45
Low arousal	3.80	1.54	3.83	1.50	3.71	1.43	3.04	= 0.05
Lockdown compliance	5.30	1.02	4.97	1.22	4.84	1.23	104.88	< 0.001
Isolation	21.37	5.04	21.54	5.13	22.04	5.12	13.94	< 0.001
Depression	5.02	5.61	5.43	6.05	5.26	5.71	5.08	= 0.025
Anxiety scale	12.92	4.56	12.9	4.53	12.78	4.63	1.56	= 0.22
PT stress					20.12	18.58		
Past focus					15.59	5.22		
Future focus					16.69	4.85		
Present focus					18.52	4.56		

a *N*_T0_=1,082 (550 women and 532 men);

*N*_T1_=786 (392 women and 394 men); *N*_T2_=632 (324 women and 308 men).

### Results and Discussion

**Figure 4:**
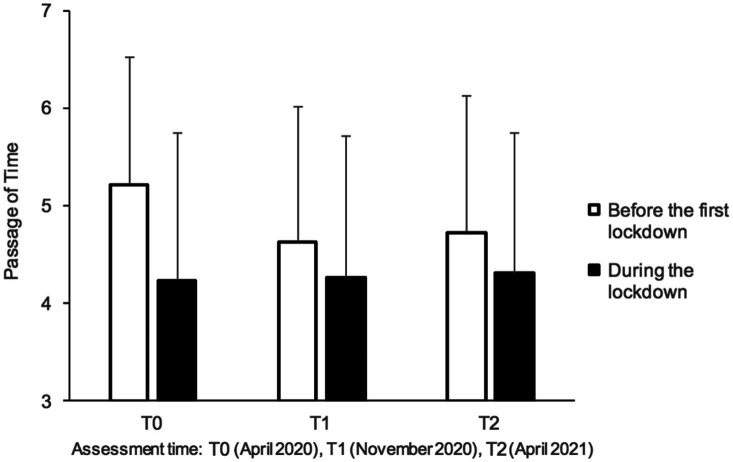
Mean ratings of passage of time for before and during the first lockdown (T0), six months after (T1) and one year after (T2).

Figure 4 shows the judgment of time experienced during the lockdown and before the lockdown at T0 (April 2020), T1 (November 2020), and T2 (April 2021). An initial analysis of variance showed no age-related effect (*p*>0.05). The main effect of sex reached significance, *F* (1, 629)=4.58, *p*=0.03, $\eta_{p}^{2}$=0.007, as did the three-way analysis for the sex×before/during×period interaction, *F* (1, 629)=4.58, *p*=0.03, $\eta_{p}^{2}$=0.007. This interaction was due to the fact that the women judged the passage of time before the lockdown to be faster (*M*=4.97, ES=0.05) than the men (*M*=4.73, *ES*=0.06), *F* (1, 630)=9.80, *p*=0.002, $\eta_{p}^{2}$=0.02, while no difference in time judgment between the women and the men was observed during the lockdown, *F* (1, 630)=0.45, *p*=0.50. However this may be, the effect size of sex was small, and this factor was therefore removed from the subsequent analyses.

The ANOVA on time judgment with two within-subjects factors (before/during, period) showed a significant main effect of before/during, *F* (1, 1,262)=159.16, *p*<0.001, $\eta_{p}^{2}$=0.20, and period, *F* (2, 1,262)=17.40, *p*<0.001, $\eta_{p}^{2}$=0.03, as well as a significant interaction between these two factors, *F* (2, 1,262)=33.65, *p*<0.001, $\eta_{p}^{2}$=0.05. This significant interaction indicated a decrease in the difference between experienced time and time remembered before the lockdown. However, this was not due to an improvement in time perception between T0 and T2, *F* (2, 1,262)=0.54, *p*=0.58, but to the decrease in the feeling that time passed faster before the first lockdown from T0 to T2, as indicated by the significant linear period effect for before-lockdown time, *F* (1, 631)=55.69, *p*=0.0001, $\eta_{p}^{2}$=0.08. In summary, the results of Study 2 replicated those of Study 1, showing that the feeling that time was passing in slow motion, already observed in the first lockdown, persisted through all three of our assessment periods without recovering significantly. They also showed that the current experience of the stretching of time during the COVID-19 pandemic “contaminated” the memory of the time flow before the first lockdown.

**Figure 5:**
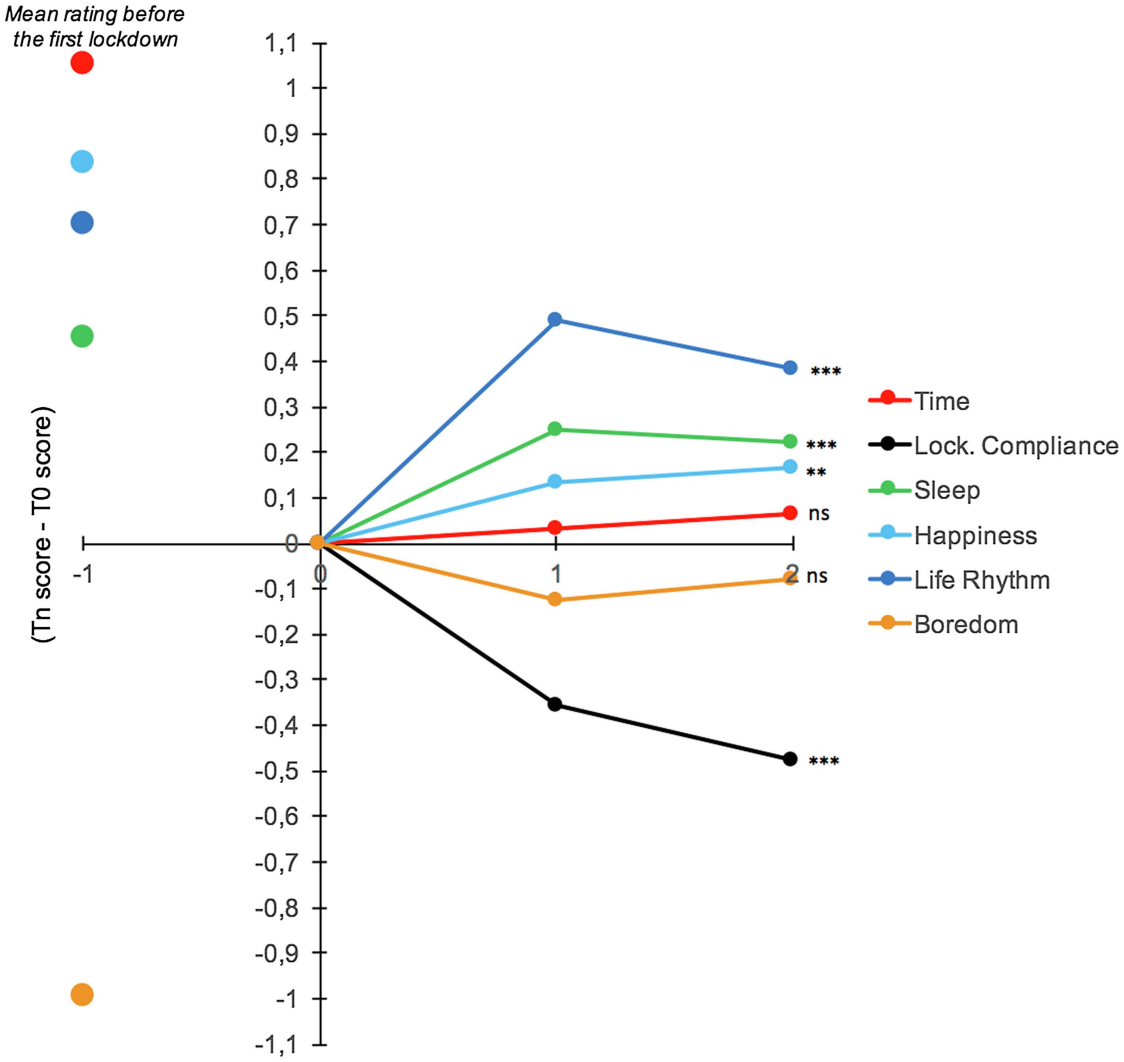
Evolution of difference index (Tn score−T0 score) for our variables of interest. The score before the first lockdown is also given as a benchmark only.

Figure 5 shows the evolution of judgments (Tn score−T0 score) of time and of the other dimensions: boredom, happiness, sleep, life rhythm, and lockdown compliance. The results of ANOVAs for each dimension with the period as within-subjects factor are reported in Table 4. There was no change in the judgment of time or the level of boredom (both *p*>0.05), with the participants having the impression that time had stopped flowing as their boredom persisted. The level of happiness nevertheless slightly increased as indicated by the significant linear *F*-value, *F* (1, 633)=9.06, *p*=0.003, $\eta_{p}^{2}$=0.014. The most important improvement was related to life rhythm, which became more regular from T0 to T2, *F* (1, 633)=27.60, *p*<0.001, $\eta_{p}^{2}$=0.04, and sleep quality, which was judged to be better, *F* (1, 633)=12.20, *p*<0.001, $\eta_{p}^{2}$=0.02. The participants were also less compliant with the lockdown measures from one lockdown to the next even though the final one was not very restrictive, *F* (1, 618)=104.88, *p*<0.001, $\eta_{p}^{2}$=0.15. However, the participants increasingly suffered from depression, *F* (1, 612)=5.08, *p*=0.025, $\eta_{p}^{2}$=0.08, and social isolation, *F* (1, 612)=5.08, *p*=0.025, $\eta_{p}^{2}$=0.08.

Table 5 shows the correlation matrix between all these variables on the difference scores between T2 and T0. As in Study 1, the changes in the feeling of time from T0 to T2 were not related to the increase in non-compliance with the lockdown measures (*R*=0.07, *p*>0.05), but to the negative emotions felt (boredom, *R*=−0.24, happiness, *R*=0.23, depression, *R*=−0.16, and isolation, *R*=−0.14, all *p*s<0.01) or the life rhythm (*R*=0.14, *p*=0.005) and sleep quality (*R*=0.14, *p*=0.008). However, the change in the passage of time took a different direction depending on the category of factors. It was experienced as passing slower as the negative affects increased from T0 to T2, and faster as life rhythm and sleep quality improved. However, a hierarchical regression analysis (Table 6) indicated that the best predictors of changes in time judgment from T0 to T2 (slower time) were related to an increase in feelings of boredom (model 4, *B*=−0.16, *ES*=0.032, *β*=−0.20, *p*<0.001) and a decrease in feelings of happiness (*B*=−0.189, *ES*=0.049, *β*=0.17, *p*<0.001). The other factors were no longer reliable predictors of changes in time when entered into the same model with boredom and happiness (Model 4). Both increased boredom and decreased happiness remained significant predictors of the increase in time dilation over the course of our assessments from T0 to T2 when included in the same model (Table 6). However, boredom explained a larger proportion of the variance than happiness.

**Table 5:** Correlation matrix in Study 2.

S. No.		1	2	3	4	5	6	7	8	9	10	11	12	13
1.	Time^a^	1	−0.24^**^	0.14^**^	0.14^**^	0.23^**^	0.07	−0.16^**^	−0.17^**^	−0.14^**^	−0.08^*^	−0.03	0.03	0.12^**^
2.	Boredom	−0.24^**^	1	−0.10^**^	0.01	−0.25^**^	−0.03	0.18^**^	0.20^**^	0.24^**^	0.10^*^	0.02	−0.04	0.01
3.	Sleep	0.14^**^	−0.10^**^	1	0.32^**^	0.27^**^	0.01	−0.16^**^	−0.19^**^	−0.13^**^	−0.04	0.01	0.01	0.02
4.	Rhythm	0.14^**^	0.01	0.32^**^	1	0.15^**^	−0.05	−0.13^**^	−0.16^**^	−0.06	−0.04	−0.02	−0.02	0.05
5.	Happiness	0.23^**^	−0.25^**^	0.27^**^	0.15^**^	1	0.10^*^	−0.16^**^	−0.20^**^	−0.20^**^	−0.05	−0.06	0.03	0.01
6.	Compliance	0.07	−0.03	0.01	−0.05	0.102^*^	1	−0.05	−0.01	−0.06	−0.01	−0.01	0.07	0.11^**^
7.	Depression	−0.16^**^	0.18^**^	−0.16^**^	−0.13^**^	−0.16^**^	−0.05	1	0.39^**^	0.29^**^	0.21^**^	0.09^*^	0.02	−0.06
8.	Anxiety	−0.17^**^	0.20^**^	−0.19^**^	−0.16^**^	−0.20^**^	−0.01	0.39^**^	1	0.26^**^	0.17^**^	0.08	−0.01	−0.09^*^
9.	Isolation	−0.14^**^	0.24^**^	−0.13^**^	−0.06	−0.20^**^	−0.06	0.29^**^	0.26^**^	1	0.17^**^	0.03	−0.03	−0.12^**^
10.	PT stress	−0.08^*^	0.10^*^	−0.04	−0.04	−0.05	−0.01	0.21^**^	0.17^**^	0.17^**^	1	0.37^**^	0.18^**^	−0.18^**^
11.	Past focus	−0.03	0.02	0.01	−0.02	−0.06	−0.01	0.087^*^	0.08	0.03	0.37^**^	1	0.39^**^	0.103^*^
12.	Future focus	0.03	−0.04	0.01	−0.02	0.03	0.07	0.02	−0.01	−0.03	0.18^**^	0.39^**^	1	0.44^**^
13.	Current focus	0.12^**^	0.01	0.02	0.05	0.00	0.11^**^	−0.06	−0.09^*^	−0.12^**^	−0.18^**^	0.10^*^	0.443^*^	1

a T2 score−T0 score for all variables except for PT stress, past, future, and present focus.

* *p*<0.05

** *p*<0.01

**Table 6:** Hierarchical regression analysis on the changes (T2score−T0 score) in the judgment of the passage of time in Study 2.

**1**	*B*	*ES*	*β*	*R* ^2^
**Model 1**				
Boredom	−0.194	0.032	−0.24^***^	0.06^***^
**Model 2**				
Boredom	−0.197	0.032	−0.24^***^	
Life rhythm	0.13	0.035	0.15^***^	
*Overall significance*		0.08^***^		
**Model 3**				
Boredom	−0.188	0.032	−0.23^***^	
Life rhythm	0.105	0.037	0.12^**^	
Sleep	0.085	0.042	0.09^*^	
*Overall significance*		0.08^***^		
**Model 4**				
Boredom	−0.159	0.032	−0.20^***^	
Life rhythm	0.093	0.036	0.11^*^	
Sleep	0.048	0.042	0.05	
Happiness	0.189	0.049	0.17^***^	
*Overall significance*		0.11^***^		
**Model 5**				
Boredom	−0.15	0.033	−0.18^***^	
Life rhythm	0.088	0.036	0.10^*^	
Sleep	0.041	0.042	0.04	
Happiness	0.193	0.049	0.16^***^	
Depression	−0.025	0.013	−0.08	
*Overall significance*		0.12^***^		
**Model 6**				
Boredom	−0.144	0.033	−0.18^***^	
Life rhythm	0.088	0.036	0.10	
Sleep	0.039	0.042	0.04	
Happiness	0.187	0.049	0.16^***^	
Depression	−0.022	0.014	−0.07	
Isolation	−0.016	0.015	−0.04	
*Overall significance*		0.12^***^		
**2**	**B**	**ES**	**β**	
**Model 7**				
Boredom	−0.192	0.031	−0.24^***^	0.6^***^
Model 2				
Boredom	−0.157	0.032	−0,19^***^	
Happiness	0.209	0.046	0.18^***^	
*Overall significance*		0.09^***^		

* *p*>0.05

** *p*>0.01

*** *p*<0.001

As in Study 1, the difference in the boredom level between T0 and T2 was significantly associated with an increase in the depression scores (*R*=0.18, *p*<0.001) and the feeling of social isolation (*R*=0.24, *p*<0.001). The participants whose depression scores increased the most from T0 to T2 were those who suffered the most from post-traumatic stress (*R*=0.21, *p*=0.001) and those who tended to be more focused on the past (*R*=0.09, *p*=0.03). No effect of sex was found for the scores on the social isolation, the post-traumatic, and the temporal focus scale (all *p*s>0.05). Only the increase in depression scores from T0 to T2 tended to be higher for the male (*M*_T2−T0_=0.86, *SD*=4.56) than for the female participants (*M*_T2−T0_=0.03, *SD*=4.96), *t* (611)=2.15, *p*=0.03, *d*=0.174. With regard to the relationship between boredom and time, the mediation analysis (Figure 2) indicated that the increase in the depression scores played a significant indirect role, although small (8.34%; *E*=−0.02, *SE*=0.007, 95%CI [−0.03, −0.003], *Z*=−2.45, *p*=0.01).

## General Discussion

We conducted a longitudinal study of two different samples of French adults. In each sample, the participants were followed up over a period of 1year: during the first (April 2020) and the third (April 2021) wave of the epidemic in France for Study 1 and during each wave (April 2020, November 2020, and April 2021) for Study 2. During each wave, people were confined to their homes but the measures were less severe from each lockdown to the next. One hypothesis was that the French population would cope with the successive lockdowns and find ways to feel better. Therefore, it was expected that the distortion of the perception of the passage of time observed in the surveys for the first lockdown (Cellini et al., 2020; Droit-Volet et al., 2020; Ogden, 2020; Martinelli et al., 2021a) would diminish over the course of the year, i.e., across the different survey periods, with a decrease in negative affects (boredom and decreased happiness) and an improvement in life conditions (life rhythm and sleep quality).

Contrary to this hypothesis, there was no or very little change in the judgment of the passage of time across the different survey periods covering an entire year, in line with the results of the recent 8-month longitudinal study conducted by Ogden (2021) in the United Kingdom. Indeed, the participants continued to experience a slowing down of time during the year (across the different survey periods), with the result that there was no improvement and no return to the initial feeling of time experienced prior to the lockdown. There was only a slight acceleration of experienced time during the time course of the first lockdown, i.e., between the beginning and the end of this long 8-week lockdown.

Confinement at home causes a rapid and radical break from the usual lifestyle. It therefore takes a transition period of several weeks to get organized and adapted. After that, this new way of life acquires a certain stability. This is corroborated by the fact that our study covering the entire period of the first lockdown showed the establishment of a more regular rhythm of life in our participants, associated with a better quality of sleep. Similarly, among a Spanish population, López-Bueno et al. (2020) observed an improvement in sleep quality after 2weeks of confinement. Consequently, our results indicated that the participants felt happier after this necessary adjustment period, even though the level of happiness remained low. More surprisingly, they still expressed a sense of boredom compared to before the lockdown. Therefore, the feeling of boredom persisted in a significant way during the entire lockdown period. More worryingly, our studies revealed that this negative affect persisted beyond the first lockdown, for the whole period of the one-year study, settling in for the long term.

The COVID-19 studies conducted on the first lockdown identified four predictors of the feeling of a slowing down of the passage of time: two linked to experienced negative emotions (increased boredom and diminished happiness) and two others to life rhythm (regularity of life rhythm and sleep quality; Droit-Volet et al., 2020; Martinelli et al., 2021a). The other factors (e.g., alcohol consumption and living space) were not reliable predictors of time judgment although all the factors were not investigated, such as daily physical activities. However, as regard this factor, French population was heavily constrained in their physical activities due to governmental regulations. The emotions of fear and anxiety about the pandemic or of anger were also not associated with the feeling of time flow in these studies. Moreover, the results of the present studies showed that these latter emotions significantly decreased with the duration of the epidemic, i.e., across our successive evaluations. Our results suggest that, as the COVID-19 epidemic has continued and lockdown periods have been reimposed, the above-mentioned two groups of factors have become in some way dissociated. Indeed, across our successive surveys, we found no or little improvement in the emotions of boredom and happiness, respectively, and a significant improvement in the life rhythm, with sleep being considered to have improved. Furthermore, our statistical analysis revealed that the only predictor that continued to be significant in our two studies was boredom (Study 1 and Study 2), although decreased happiness also had an impact, albeit smaller (Study 2). The other predictors related to life rhythm were no longer significant. The feeling that the passage of time had stopped or was stretching out forever was therefore rooted in a persistent negative mood, despite some lifestyle improvements. The originality of our findings is therefore to show that emotions are at roots of changes in perception of the passage of time during the lockdown periods.

Numerous studies conducted in different countries have observed a significant increase in depression scores with the COVID-19 lockdown (Bueno-Notivol et al., 2021), and this is a characteristic of psychological states observed during each lockdown or period of quarantine (Henssler et al., 2020). Our results indicated that these depression scores, which were already high during the first lockdown, were even higher a year later (see also Ogden, 2021). The increase in the depression scores from April 2020 to April 2021 also appeared to be significantly related to scores on the post-traumatic scale. In addition, the more traumatized people were, the more they focused on the past. This is consistent with earlier studies on traumatic situations and the temporal perspective (Zimbardo and Boyd, 1999; Holman et al., 2016) as well as with the most recent studies on the first lockdown (Sobol et al., 2020; Virna and Brahina, 2020; Wu et al., 2021). However, no sex effect on temporal perspective was observed in our French population, unlike in Sobol et al.’s (2020) or Virnal and Brahina’s (2020) studies. The mean age of our final sample, i.e., the participants who completed the last survey, was reasonably high (*M*_study1_=44.65, *SD*=16.38; *M*_study2_=49.22, *SD*=14.31), and age and associated economic status are known to be protective factors against depression (e.g., Freeman et al., 2016). In line with the studies on emotional functioning across adulthood (Carstensen et al., 2000; Bruine de Bruin et al., 2016), the COVID-19 studies have indeed found higher depression scores in younger people (Martinelli et al., 2021b). With a younger sample of participants, our results might perhaps have been characterized more by a similar sex effect. Our sample is nevertheless more representative according to age than previous studies on time. However that may be, the depression scores assessed in our studies significantly mediated the effect of boredom on the feeling of time. The judgment of time flow in daily life is thus highly sensitive to emotional state and depressive mood. Time judgment is therefore a good – and easy-to-measure – indicator of psychological health. However, time judgment assessed in our study was only one component of time judgment which encompasses different subjective time experiences, like interval duration estimation or the perceived speed of time passage as that examined in our studies (see Loose et al., 2021). In sum, our studies on this specific component of time judgment have shown that, as of the first lockdown, individuals have experienced no further variation in the speed of the passage of time, which has always been found to have stagnated or to be flowing slowly. This experience of time was found to be significantly related to the feeling of boredom, associated with a depressive state that tended to be chronic.

Our results indicate that the increase in depression scores between April 2020 and April 2021 had a significant mediating effect on the relationship between changes in boredom and time judgment. However, this mediating effect remained small (15.1% Study 1; 8.34% Study 2). The direct effect of boredom on time remained highly significant. As explained in contextual self-duration theory (Martinelli and Droit-Volet, 2021; Droit-Volet and Martinelli, in press), the judgment of the passage of time is highly dependent on the life context. We can therefore assume that the feeling of slowed-down time and its main predictor – boredom – were also related to the context; i.e., the time our survey was performed. Our surveys were always performed during a lockdown period. In other words, the feeling of slow time and considerable boredom might have persisted because confinement at home persisted. This is likely to prove to be the case and we can hope for a return to normal with the development of collective immunity and the end of the lockdown periods. That being said, our data also revealed that the participants were less compliant with the lockdown rules from one lockdown to the next and that this behavior did not contribute to changes in the experience of time and the level of boredom. In addition, and as reported earlier in this paper, the context of the French lockdown on April 2021 was not highly constraining and some people even questioned the reality of confinement. In spite of the flexibility of the last confinement and the improvement in the life rhythm, boredom and the feeling of a slow passage of time persisted. This is consistent with the idea that the judgment of the passage of time results from the conscious analysis of our emotional state, something which goes beyond the immediate life context (Droit-Volet and Dambrun, 2019; Droit-Volet and Martinelli, in press). Put differently, this judgment, like many others, is based on psychological representations that do not always correspond to reality.

## Conclusion

In conclusion, our longitudinal research indicates that the French people have the feeling that time has been dragging on at a slow pace since the beginning of the first lockdown and that it has not resumed its normal course. This is explained by the establishment of a persistent sense of boredom which is characteristic of a chronic depression that has taken hold in the population. However, the limits of our studies on time are to have not further examined the individuals’ traumatic experiences, clinically evaluated their depression or collected information on their depression history (medication intake, etc.). Nevertheless, our studies clearly showed that the repeated contexts of confinement did not improve individual time judgment. Our research needs to continue, and we need to undertake further assessments to see whether, how and when individuals return to a more psychologically healthy state, which would be evidenced by an acceleration of psychological time.

## Data Availability Statement

The raw data supporting the conclusions of this article will be made available by the authors, without undue reservation.

## Ethics Statement

The studies involving human participants were reviewed and approved by the Research Ethics Committee of the University Clermont Auvergne (IRB00011540-2020-31). The participants provided their written informed consent to participate in this study.

## Author Contributions

SD-V, NM, CB, SG, and PH conceived the survey. NM collected and analyzed the data. SD-V analyzed the data and drafted the manuscript. SG, CB, GD, JC, and PH provided critical revisions and approved the final version of the manuscript. PH and SD-V secured funding for the study. All authors contributed to the article and approved the submitted version.

## Conflict of Interest

The authors declare that the research was conducted in the absence of any commercial or financial relationships that could be construed as a potential conflict of interest.

## Publisher’s Note

All claims expressed in this article are solely those of the authors and do not necessarily represent those of their affiliated organizations, or those of the publisher, the editors and the reviewers. Any product that may be evaluated in this article, or claim that may be made by its manufacturer, is not guaranteed or endorsed by the publisher.
